# Effective intravenous immunoglobulin therapy for Churg-Strauss syndrome (allergic granulomatous angiitis) complicated by neuropathy of the eighth cranial nerve: a case report

**DOI:** 10.1186/1752-1947-6-310

**Published:** 2012-09-18

**Authors:** Yoshio Ozaki, Akihiro Tanaka, Keiko Shimamoto, Hideki Amuro, Yonsu Son, Tomoki Ito, Shosaku Nomura

**Affiliations:** 1 Department of Rheumatology and Clinical Immunology, Kansai Medical University Hirakata Hospital, 2-3-1 Shin-machi, Hirakata City, Osaka, 573-1191, Japan; 2 First Department of Internal Medicine, Kansai Medical University, 10-15 Fumizono-cho, Moriguchi City, Osaka, 570-8506, Japan

**Keywords:** Churg-Strauss syndrome, Cranial neuropathy, Intravenous immunoglobulin

## Abstract

**Introduction:**

We report the case of a patient with Churg-Strauss syndrome with eighth cranial nerve palsy. Vestibulocochlear nerve palsy is extremely rare in Churg-Strauss syndrome. To the best of our knowledge, only one case of complicated neuropathy of the eighth cranial nerve has been described in a previous report presenting an aggregate calculation, but no differentiation between polyarteritis nodosa and Churg-Strauss syndrome was made. High-dose immunoglobulin was administered to our patient, and her neuropathy of the eighth cranial nerve showed improvement.

**Case presentation:**

At the age of 46, a Japanese woman developed Churg-Strauss syndrome that later became stable with low-dose prednisolone treatment. At the age of 52, she developed sudden difficulty of hearing in her left ear, persistent severe rotary vertigo, and mononeuritis multiplex. At admission, bilateral perceptive deafness of about 80dB and eosinophilia of 4123/μL in peripheral blood were found. A diagnosis of cranial neuropathy of the eighth cranial nerve associated with exacerbated Churg-Strauss syndrome was made. Although high doses of steroid therapy alleviated the inflammatory symptoms and markers, the vertigo and bilateral hearing loss remained. Addition of a high-dose immunoglobulin finally resulted in marked alleviation of the symptoms associated with neuropathy of the eighth cranial nerve.

**Conclusions:**

A high dose of immunoglobulin therapy shows favorable effects in neuropathy of the eighth cranial nerve, but no reports regarding its efficacy in cranial neuropathy have been published.

## Introduction

Churg-Strauss syndrome (CSS), also known as allergic granulomatous angiitis, is a syndrome consisting of angiitis in the systemic small vessels, bronchial asthma, eosinophilia, fever, and vasculitis of various organ systems [1]. Mononeuritis multiplex is seen in 36.1% to 71.8% of cases of CSS [2-5]. However, cranial neuropathy is rare in CSS, and neuropathy of the eighth cranial nerve is extremely rare. Although a case of complicated neuropathy of the eighth cranial nerve was described in a previous report presenting an aggregate calculation without differentiating between polyarteritis nodosa and CSS [6], no cases that could be definitively diagnosed as CSS have been reported.

Intravenous infusion therapy with high doses of immunoglobulin (intravenous immunoglobulin, or IVIg) has been shown to be efficacious in Kawasaki disease and idiopathic thrombocytopenia. Also, IVIg was reported to be effective in cases of new-onset CSS [7] and in pregnant women with CSS in whom immunosuppressants cannot be administered [8]. In Japan, IVIg has been approved for neuropathy of the peripheral nerves in CSS as coverage under the national health insurance scheme. Although IVIg shows favorable effects in neuropathy of the peripheral nerves, no reports regarding its efficacy in cranial neuropathy have been published. Here, we report a case of CSS complicated by neuropathy of the eighth cranial nerve in which IVIg was effective.

## Case presentation

A 35-year-old Japanese woman with no notable medical or family histories developed bronchial asthma. Treatment was initiated, and the course of bronchial asthma had been favorable. After 11 years, rotary vertigo, difficulty of hearing in the right ear, and palsies of the left lower extremity and right forearm occurred. A diagnosis of CSS was made on the basis of bronchial asthma, neuropathy of the peroneal nerve and ulnar nerve, findings of angiitis by biopsy of the left sural nerve, increased eosinophil counts, and positivity for myeloperoxidase anti-neutrophil cytoplasmic antibody (MPO-ANCA). The pathological conditions were alleviated by prednisolone (PSL) treatment with a tapering schedule from a dose of 1mg/kg and methyl-prednisolone pulse therapy (mPSL). Three years later, owing to another increase in MPO-ANCA and exacerbation of peripheral nerve neuropathy during PSL treatment at a dose of 10mg/day, mPSL pulse therapy was administered again and the dose of PSL was increased to 50mg/day (1mg/kg). However, because the neuropathy was not alleviated, intravenous cyclophosphamide (IVCY) pulse therapy (750mg/body) was administered six times every four weeks. The IVCY therapy stabilized our patient’s condition. Subsequently, the dose of PSL was decreased to 10mg/dL gradually.

More than three years later (when our patient was 52 years old), she was hospitalized again because of the recurrence of bilateral hearing difficulty and rotary vertigo. At admission, physical findings included a body temperature of 36.2°C, a blood pressure of 132/74mmHg, a pulse of 66 beats per minute, and a respiratory rate of 18 per minute. No abnormal cardiac sounds were noted, and mild wheezing sounds were present in the lung field. Spontaneous leftward horizontally rotary mixed nystagmus was present, and owing to strong vertigo our patient had difficulty being in a sitting position. A hearing test revealed average hearing thresholds of 86.3dB in the right ear and 78.8dB in the left ear, which indicated severe perceptive deafness (sensorineural hearing impairment). She did not have tinnitus or recruitment phenomenon. Neurological findings included neuropathies of the right ulnar, left peroneal, and right tibial nerves, and left foot drop. Chest radiography and head magnetic resonance imaging did not show any abnormalities, and examination findings included a leukocyte count of 13,300/μL, an eosinophil count of 31.0% (4123/μL), a C-reactive protein (CRP) level of 6.408mg/dL, and an MPO-ANCA of 226EU (Table 1). The results of serum cytokine measurements by the cytometric bead array method were as follows: interleukin-4 (IL-4) of 3.3pg/mL (normal is less than 1.4), IL-5 of 11pg/mL (normal is less than 1.1), IL-6 of 6.9pg/mL (normal is less than 1.6), IL-10 of 4.9pg/mL (normal is less than 0.13), IL-13 of 7.4pg/mL (normal is less than 0.6), and interferon-gamma (IFNγ) of 0.1pg/mL (normal is less than 1.8).

**Table 1:** Laboratory findings of the patient on admission

**Parameter**	**Value**	**Normal range**
White blood cell count, ×102/μL	133	35-85
Neutrophil, percentage	63.0	42.0-77.0
Basophil, percentage	0.5	0.1-2.0
Eosinophil, percentage	31.0	0.5-6.0
Lymphocyte, percentage	3.5	18.0-49
Monocyte, percentage	2.0	3.0-9.0
Red blood cell count, ×104/μL	413	370-510
Hemoglobin, g/dL	14.5	11.3-15.4
Hematocrit, percentage	41.9	34 - 46
Platelet, 104/μL	18.1	14 - 34
Glucose, mg/dL	98	60-100
Hemoglobin A1c, percentage	5.7	4.0-6.0
Sodium, mEq/L	144	138-146
Potassium, mEq/L	3.7	3.5-5.0
Chloride, mEq/L	102	100-110
Blood urea nitrogen, mg/dL	8	8-20
Creatinine, mg/dL	0.55	0.4-0.8
Uric acid, mg/dL	3.1	2.5-5.5
Calcium, mg/dL	8.8	8.5-10.3
Total protein, g/dL	6.2	6.5-8.2
Albumin, g/dL	3.9	3.8-5.0
Aspartate aminotransferase, U/L	17	13-35
Alanine aminotransferase, U/L	5	5-35
Total bilirubin, mg/dL	0.6	0.2-1.2
Alkaline phosphatase , U/L	115	107-340
γ-Glutamyl transpeptidase, U/L	36	8-45
Lactate dehydrogenase, U/L	226	112-230
Creatine kinase, U/L	36	45-165
Amylase, U/L	61	37-125
C-reactive protein, mg/dL	6.408	<0.3
IgG, mg/dL	883	870-1700
IgA, mg/dL	112	110-410
IgM, mg/dL	82	46-260
IgE, IU/mL	421	<320
MPO-ANCA, EU	226	<20
PR3-ANCA, U/mL	0	<3.5
Rheumatoid factor, IU/mL	5	<20
Anti-nuclear antibody	×20	<×40

Ig: immunoglobulin; MPO-ANCA: myeloperoxidase anti-neutorophil cytoplasmic antibody; PR3-ANCA: proteinase 3 anti-neutorophil cytoplasmic antibody.

The diagnosis was neuropathy of the eighth cranial nerve associated with exacerbation of allergic granulomatous angiitis, and mPSL pulse therapy and subsequent oral PSL at a dose of 55mg/day (1mg/kg per day) were begun. This treatment resulted in gradual alleviation of bronchial asthma, eosinophilia, CRP level, and mononeuritis multiplex but not of the foot drop. Also, the hearing difficulty and vertigo were not alleviated. Although reinforced treatment by IVCY was planned initially, our patient refused because of the severe nausea that occurred in the previous IVCY treatment. Therefore, IVIg (400mg/kg per day for five days) was combined from day 14. From around day 20, the hearing ability and foot drop began to improve gradually. On day 42, a hearing test revealed average hearing thresholds of 70.0 and 61.7dB in the right and left ears, respectively, and the vertigo had disappeared. After our patient was discharged from the hospital, the dose of PSL was decreased to 10mg/day and her condition is currently stable.

## Discussion

Because the vertigo continued for a long period of time and recruitment phenomenon and tinnitus were not present, we ruled out atypical sudden deafness in our patient. Since auditory brain-stem response was not performed, the cause of her hearing loss was not identified. Her vertigo and deafness were likely the results of CSS exacerbation or late flare-up, which was evidenced by rising titers of MPO-ANCA and inflammatory markers. Thus, the presumptive diagnosis of CSS-associated cranial neuropathy involving the eighth cranial nerve was made, and the condition subsequently resolved with immunoglobulin therapy.

## Conclusions

CSS accompanying cranial neuropathy is relatively rare. Guillevin *et al*. [9] reported that only four out of 96 CSS cases were complicated by cranial neuropathy. In the present case, the perceptive deafness with vertigo corresponded to pathological conditions of CSS, such as increased MPO-ANCA and some inflammatory markers and exacerbated neuropathy of the peripheral nerves. With regard to serum cytokine concentrations in CSS, increases in Th2 cytokines IL-4 [10], IL-5 [11], IL-10 [12], and IL-13 [10,, 13] have been reported, although there are reports showing no apparent increases in IL-4 [13], IL-5, and IL-10 [10]. In the present case, increases in serum level of inflammatory cytokine IL-6 and IL-4, IL-5, IL-10, and IL-13, but not Th1 cytokine IFNγ, were found.

As a disease complicated by angiitis and perceptive deafness, Cogan syndrome in aortitis syndrome is well known. However, the pathogenesis of perceptive deafness with Cogan syndrome remains unclear. Meanwhile, the etiology of neuropathies in CSS has been suggested to be involved in damage by feeding vessels of the peripheral nerves.

It is speculated that the effect of IVIg is due to the blockade of Fc receptors, adjustment of complement activities with idiotype network [14], or inhibition of damage to myelin or axons as an antigen [15]. In the present case, as for mononeuritis multiplex of the sensory nerves, mPSL pulse therapy was effective. The foot drop as a neuropathy of the motor nerve and the neuropathy of the eighth cranial nerve were resistant to the mPSL pulse therapy but were alleviated after administration of IVIg. We inferred that the effects of steroids were enhanced through some active mechanisms of IVIg as speculated above. Given our present case, IVIg could possibly be a useful treatment for cranial neuropathy associated with CSS and should be taken into consideration for cases in which the effects of mPSL pulse therapy are insufficient or administration of immunosuppressants is difficult.

## Consent

Written informed consent was obtained from the patient for publication of this case report and any accompanying images. A copy of the written consent is available for review by the Editor-in-Chief of this journal.

## Abbreviations

CRP: C-reactive protein; CSS: Churg-Strauss syndrome; IFNγ: interferon-gamma; IL: interleukin; IVCY: intravenous cyclophosphamide; IVIg: intravenous immunoglobulin; MPO-ANCA: myeloperoxidase anti-neutrophil cytoplasmic antibody; mPSL: methyl-prednisolone pulse therapy; PSL: prednisolone.

## Competing interests

The authors declare that they have no competing interests.

## Authors’ contributions

YO wrote the paper. SN helped to write and revise the paper. KS and TI performed the cytokine analysis. YS, HA, and AT took responsibility for the clinical management of our patient. All authors read and approved the final manuscript.
